# Pre-Planting and Planting Factors and Practices Affecting Urban Tree Growth: With a Special Focus on the Root System and Its Condition—A Review

**DOI:** 10.3390/plants14030387

**Published:** 2025-01-27

**Authors:** Mateusz Korbik, Tatiana Swoczyna, Piotr Latocha

**Affiliations:** Department of Environmental Protection and Dendrology, Institute of Horticultural Sciences, Warsaw University of Life Sciences, 02-776 Warsaw, Poland; mateusz_korbik@sggw.edu.pl (M.K.); tatiana_swoczyna@sggw.edu.pl (T.S.)

**Keywords:** tree planting quality, planting issues, root pruning, maintenance after planting

## Abstract

Trees in urban conditions struggle with many factors that reduce their growth. In many cases, newly planted trees do not survive to maturity. The trees are produced using various methods, the most popular of which are balled and burlapped (B&B) and container production. Different production methods have their cons, but in many cases, the most common problem is the root system condition—it is often poorly developed, with girdle roots, or the rootball is covered with excess soil. Deep structural roots, as this is the name of the problem related to the roots being located too deep in the soil during production or trees being placed too deep in the planting pith, have been noticed for several decades; nevertheless, they are still poorly understood. In many cases, the excess soil above the rootball is over 10 cm—such covering the rootball may lead to infection, weakening, or tree death. The problem of deep structural roots seems to be one of the most serious problems we face in the case of urban plantings. However, many other factors remain disputed—such as cutting the crowns of planted trees, removing burlap from a rootball, or planting smaller rather than larger trees. All these issues have not been resolved despite many years of study, and still require further investigation.

## 1. Introduction

The intensive development of cities combined with climate change is drastically deteriorating living conditions. Therefore, trees in cities are increasingly appreciated because of their ability to minimize these unfavorable changes [1,2]. Even the widely known 3–30–300 rule connected to health and well-being benefits directly relates to trees—making them one of the main components of the concept [3]. Unfortunately, newly planted urban trees must withstand harsh conditions, which constantly worsen with climate change, ongoing development, and city inhabitants’ activity [4]. Proper management of urban greenery should ensure the monitoring and continuity of urban afforestation through effectively planting new trees [2,5,6,7]. Many factors occurring at different stages of tree lifespan appear to influence tree establishment and health significantly. However, it is said that the initial 1–5 years after planting are the most crucial, when the mortality of trees is the highest [8]. In recent years, studies on urban tree mortality have increased significantly. According to cohort studies, the annual mortality of newly planted urban trees can reach up to 70%; at the same time, for inventories repeated for trees of uneven age, annual mortality can reach up to 30%. The mentioned studies indicate that the half-life for urban trees planted in poor-condition sites is around 7 to 11 years, which signals a serious problem with young planting in urban areas [9]. These numbers illustrate that young trees deal with problems that lead to their hastier death. Numerous factors like drought, soil pollution, high temperatures, and others are responsible for this and are progressively worsening [10]. Still, some tree plantings with high establishment survival are worth monitoring and emulate the best management practices [11].

The complex process of tree production and planting has many issues at every stage. There are no significant differences if professionals or trained non-professionals plant trees. Site conditions may impact tree mortality, but species selection, production method, stock quality, planting process, and post-planting tree care are of greater significance [8,12,13,14]. The influence of nursery production systems on urban tree survival has already been studied, especially by Whitcomb [15], Day et al. [16], and Allen et al. [17], indicating numerous connections between nursery production, planting practices, and post-transplant tree survival. Nevertheless, nursery production and additional factors connected to tree performance and survival still need more attention. Due to insufficient research covering these issues, not only does the recent literature need to be reviewed, but also older studies, which will help to fully understand the progress and direction in which our understanding of tree planting heads.

Some of the main abiotic stress factors that influence young urban trees’ conditions are (1) soil degradation, i.e., compaction, drought, extreme soil temperatures, contamination, pH changes, and nutrient deficiency; (2) light condition disturbances, i.e., inadequate doses, light pollution; (3) air quality, namely, air temperature, water vapor deficit, and pollution [10,12,18,19,20]. Poor site conditions can be deadly for trees or at least lead to reduced growth and health [21]. Temperature increases and drought associated with climate change make urban heat islands an even more serious threat to species used [13,22,23,24,25]. A large number of taxa is used in city plantings, although only a few as street trees in many central and northern European countries—where about 50–70% is represented by 3–6 genera [26,27], and in some individual cases over 50% by just a single-clone, like *Tilia* × *europaea* L. ‘Pallida’, which makes about 70% of newly planted trees in Oslo, Norway [26]. Even though studies show that some trees, like *Acer platanoides* L. or *Tilia* spp., are very sensitive to salinity stress and drought, they are still widely planted as street trees [28,29,30,31,32,33]. Other species often planted in urban areas belong to the genera *Aesculus* L., *Fraxinus* L., *Platanus* L., and *Quercus* L. [25], including some species suitable for street planting [32,34]. Knowledge about conditions at the planting site and choosing suitable species is fundamental; nevertheless, high-quality stock is also required for successful tree establishment [35]. Poor-quality stock is still being sold, and incorrect planting techniques are being used, making it hard to establish young trees in the harsh urban environment [36]. The urban environment conditions are genuinely challenging for trees. Therefore, problems of nursery stock preparation for planting and issues concerning the planting process must be fully understood to identify the weakest points in those processes and develop best practices for further tree care after planting and establishment. This paper reviews the nursery practices and planting recommendations discussed in the last five decades of urban afforestation. The tree production and pre- and post-planting practices were considered, with special attention given to identifying factors influencing the tree’s establishment and development in the first years after planting.

## 2. Characteristics of Different Types of Nursery Stock

Due to the growing demand for trees over the last several decades, nursery production has changed significantly, leading to production automation, which appears to be widespread currently [37]. Equipment such as tree spade transplanters have been used for decades. Also, devices influencing the condition of the plants are used more often, which leads to the production of higher-quality material [38,39]. Tree production can be divided into container and field-grown, where the container production system includes traditional plastic, metal, wood, or fabric containers, pot-in-pot containers, in-ground fabric containers, and field-grown trees, including bare-root, rootball-excavated, and burlap-wrapped trees [17,40,41]. Each production method and technology has its advantages and disadvantages, affecting both production costs and the quality of plant material [42,43]. Statistically, the production method significantly impacts the post-planting tree survival rate [8]. This is why it is so essential to understand what problems trees cope with at a nursery. All trees should be transplanted during production to develop a proper root system, using a tree spade or manually moving them into a larger container. During that process, trees are transplanted usually every 2–3 years—if not, it should be performed at least every 5 years [44]. According to Watson and Hewitt [45], nursery-produced trees have 7–48% fewer roots, which can develop into proper root flares compared to untreated rootballs. Day et al. [19] noted that a healthy root system influences tree performance and improves soil condition. Thus, root system formation and protection methods should be considered when planting trees under challenging sites.

### 2.1. Trees with Balled and Burlapped Root System (B&B)

After harvesting, the tree’s rootball is wrapped with burlap, a biodegradable material made of jute or hemp that does not let the rootball fall apart and protects roots from drying during transport. After wrapping with burlap, the wire basket is placed around the rootball and tightened using a drill with a hook; this helps keep the rootball’s shape and makes it easier to transport and plant the tree [15]. Following the harvesting, roots should be regenerated within a rootball. Recently dug trees are supposed to be watered and stay in the nursery for at least 10 weeks before shipping; this leads to a high survival rate after planting [46]. Roots regenerate most efficiently between 10 °C and 20 °C [47]. It may take about 7 weeks to develop new roots after pruning the rootball and about 20 weeks to start absorption in sufficient amounts [48]; therefore, letting the roots regenerate before the tree is harvested is essential. Among the methods mentioned above, so far, burlap-wrapped trees have been the most commonly used in the landscaping industry, with about 90% of trees produced this way planted in urban areas in the northeastern United States [43] and almost 100% out of the thousands planted street trees in the most recent few years in Warsaw, Poland (Warsaw Municipal Greenspace Authority, unpublished information). The number of transplants during nursery production should be identified as every single transplant starting from the moment when the tree is placed at the nursery in a particular propagation site (applies to trees propagated from seed, cuttings, and stool beds as well), and ending with digging out a tree to transport it to the place where it will be eventually planted [44]. Due to the transplant, roots are pruned each time, creating a denser root system within the rootball, made of numerous regenerated adventitious roots at the cut end [49]. Studies by Miller and Graves [50] showed that pruning increased the number of roots of different *Carya* spp. seedlings, and applying auxin significantly increased the total number of roots by ca. 79% to 152%. Moreover, according to Harris et al. [51], proper pruning during the first two transplants significantly increases the percentage of trees with roots free from defects. Root pruning and regeneration make the root system proportional to the crown’s size created during production [52]. Proportionality of the below- and aboveground biomass may be recognized by some oppositely, i.e., as the need for a strong pruning of the crown to equate it with a strongly reduced root system, usually due to incorrect production at the nursery, but not as it should be, i.e., as creating an appropriate density of roots that can supply water and minerals to the well-developed crown [15,48]. A properly developed root system is essential, especially for trees planted into scantily provided sites, where trees most likely never build back proper root–shoot stability [53].

The role of proper production of trees with balled and burlapped root systems is significant. Correct transplanting and pruning of the roots lets the tree create a dense root system due to the process, which can supply it with water after planting when it is needed the most. Availability of water is one of the most important factors affecting the establishment of new plantings [54,55], especially in harsh urban conditions, where it takes a transplanted tree about 10 years to fully regrow the root system reduced while replanting [56,57]. Due to the last transplant before planting in the final destination, the reduction in the root system may reach about 95–98% of its total volume [54,58]. Usually, less than 5% of the root system is transplanted with the tree, which may lead to serious water management problems.

According to Day et al. [19], about 40–73% of assimilated carbon is directed below the ground, indicating the importance of losing a large part of the root system. Nevertheless, regular pruning of the root system may increase its area by four times compared to the root systems that are not pruned [52]. Also, the volume of the rootball of a field-grown tree can be twice that of a container-grown tree [59]. The production method significantly affects the quality of a tree. According to studies, the survival rate of field-grown burlap-wrapped trees in conditions with limited availability of water is higher (86%) than the survival rate of container-grown trees (45%), which die sooner and more often, while for trees irrigated regularly and maintained properly in the first year after planting, the production method seems not to have any significant impact [46,59,60]. Regular watering is one of the most important tree maintenance activities [8,61,62].

The availability and diversity of burlap-wrapped trees are much more significant than other production methods and encompass various species, cultivars, or a wide range of tree sizes [63]. Burlap-wrapped trees usually perform very well, having a strongly developed and dense root system if produced correctly [52]. According to European Tree Planting Standard [64], the best time to plant B&B trees is autumn to spring. This kind of material also reveals some disadvantages. Particularly, the weight of B&B trees can make them more problematic to transport and plant, usually performed with a loader crane and skilled personnel [15,63]. Nevertheless, because of the rootball, which protects roots from drying, trees produced this way handle transportation better and can be stored a little longer on the planting site before the planting. Because of heavy rootballs, burlap-wrapped trees may require staking sometimes [41]. Gilman noticed that seven months after planting, B&B trees were rooted and anchored to the soil about 50% better than container trees [65]. Due to the advantages mentioned and additional transport and planting costs, B&B trees are also characterized by high prices [17,43].

**Figure 1:**
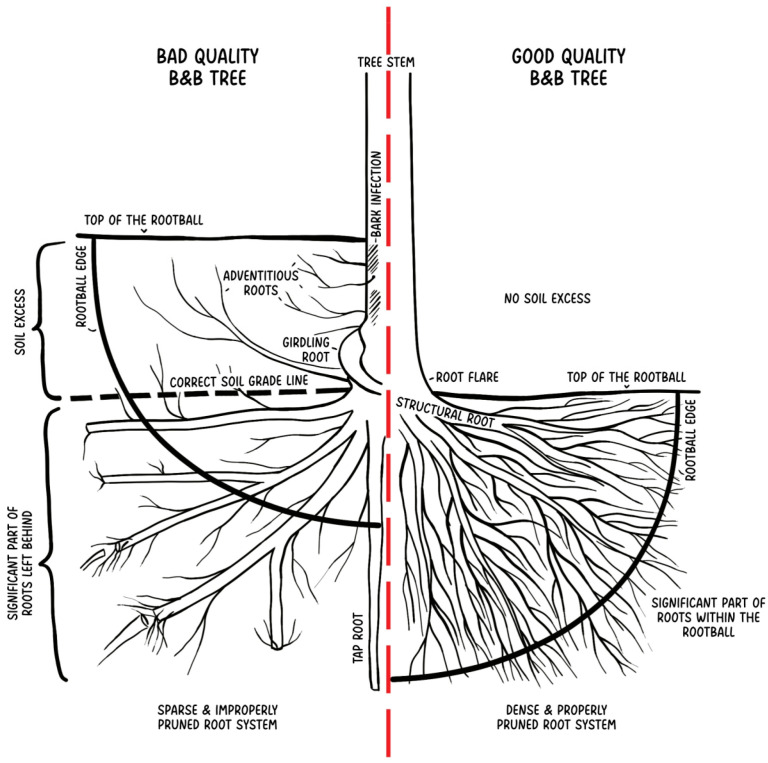
Comparison of bad- and good-quality B&B trees.

During the automated replanting process, trees at a nursery may be covered with an additional layer of soil and grow this way until they are dug up and burlap-wrapped. A rootball of a tree with deep structural roots is often undersized, related to the harvesting process, which includes digging into a certain depth. Due to that process, the extra layer of soil above the structural roots is usually not considered. Because of the extra soil layer on top of the rootball, the lower part of the root system is cut off by a spade and left at the growing site [49]. It is confirmed that trees with deep structural roots can have significantly fewer topsoil roots than properly transplanted ones [66]. It is problematic because rainfall and mineral nutrients are the most accessible near the surface [67]. This is where the roots are the most needed, especially after transplanting. A bad-quality B&B tree can be characterized by the sparse and improperly pruned root system, with a significant part of the roots left behind when dug out—this is caused by the improper digging depth, which leaves the lower part of the roots in the ground. Soil excess may lead to bark infections and adventitious roots development; girdling roots may occur. A good-quality B&B tree is characterized by visible root flare and structural roots near the surface; the root system is dense, with many roots within the rootball (Figure 1).

### 2.2. Container Trees

Production of container-grown trees has been increasing due to the demand for high-quality plants all year, with millions of trees and shrubs sold worldwide each year for a few decades [49,68,69,70]. In this type of production, trees grow in containers all the time, and, unlike burlap-wrapped trees, they are not transplanted by machines like tree spades, damaging the root system. However, they are replanted into a larger container that provides more space for further root system growth [15]. Because of that, root loss in container-grown trees is not as significant as in burlap-wrapped trees when transplanted to the final destination [17]. Usually, container-grown and burlap-wrapped trees have the same volume of roots within the rootball after harvesting [59,71]. In container production, each time the roots and soil are transplanted into a larger container, the root system stops growing in length upon contact with air or water; after that, the root tip dies and branches out [49]. The undoubted advantage of this treatment method is the availability of trees all year round due to requirements for digging out and being burlap-wrapped [64]. Container-grown trees are much lighter than burlap-wrapped trees [41] and, therefore, easier and cheaper to transport and plant [17].

Due to the different nature of the growth of the root system, which is limited by the presence of the container, improper replanting can result in deflected or circling roots, which are common defects of this kind of nursery stock [70,72,73,74]. Fare [75] noticed that trees planted 15 cm too deep in the container had many circling roots. Even if proper root pruning can reduce defects, correct transplanting at the right time is still necessary [51]. Different species are characterized by different growth of roots; some produce just a few main roots (usually one or two), while others produce numerous, more fibrous roots [76]. This makes some tree species more likely to develop defective root systems during production. Circling roots may turn into girdling roots when close enough to the trunk, which may impact the tree’s condition in the future. It is essential to check the root system and cut through the rootball if any circling roots are present; at the same time, girdling roots should be cut as soon as possible. Container trees with defective root systems may require the removal of all roots located above structural roots, cutting off all girdling roots even if they are structural roots, and shaving the rootball from all sides by cutting off the peripheral part at least 2.5 cm deep [77,78,79]. Shaving may also improve rooting and tree anchorage to the soil by about 13% [65,80]. Struve [81] and Appleton [82] indicate that trees produced in copper-treated containers usually do not need root pruning and have a lesser tendency to develop girdling roots; there are also numerous different types of containers like modified “low profile”, “soil sock”, or “pot-in-pot”, which reduce that problem.

**Figure 2:**
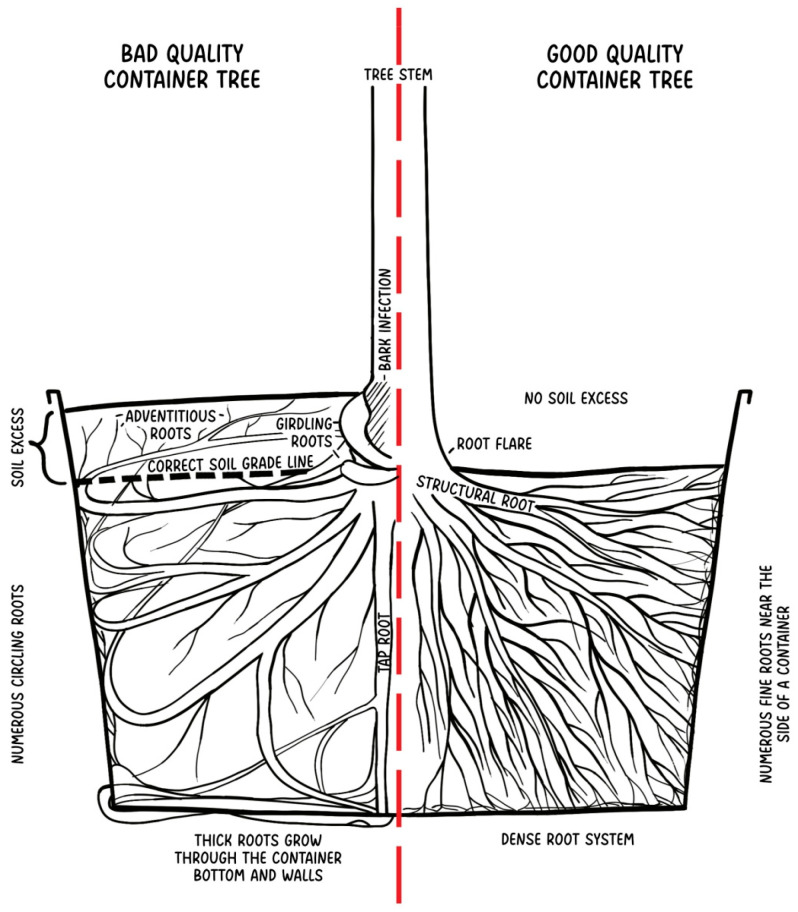
Comparison of bad- and good-quality container trees.

Another common problem similar in both burlap-wrapped and container trees is deep structural roots. Due to the production process, container trees are replanted and are usually overburdened, each time more. Some producers practice covering them with extra soil to hide cutback wounds or a graft union, even if these are not supposed to be treated as defects and should be visible [49]. Trunk distortion at the grafting point, sometimes present, disappears later when the trunk grows [75]. According to some nursery catalogs, the proper planting depth for peach trees was 35 cm deeper than they were growing in the nursery [83]. Some believed that deep planting provides better stability without the need for staking [84]; this was quickly denied and it was stated that due to poor growth of deeply planted trees, they are supposed to be planted no more than 2–5 cm above the first structural roots [83]. Despite this, liners are still occasionally planted too deep in the containers to keep them in place during the transport [75]. Tree roots stored in containers throughout the winter are also more likely to suffer from frost damage [85]. Unfortunately, there is less knowledge and less attention given to container-grown plants and their deep-root architecture than field-grown trees sold with bare roots or burlap-wrapped [46,49,61,70]. What is interesting is that container-grown trees are characterized by weaker growth [86] and worse establishment than burlap-wrapped trees if not irrigated [46]; otherwise, their overall mortality seems to be similar to B&B trees [8]. A bad-quality container tree can be characterized by numerous circling roots near the container wall, which may often lead, together with soil excess, to girdling roots development around root flare; roots can grow through the container bottom and walls. Bark infections may occur. A good-quality container tree has a visible root flare with a very dense root system with numerous small roots near the walls of the container but without pronounced circling roots (Figure 2).

### 2.3. Bare-Root Trees

The least popular planting material is bare-root trees. Nevertheless, they are still sold in many countries in hundreds of millions of specimens [87]. They are produced in the field, dug up similarly to burlap-wrapped trees, but without forming and wrapping the rootball. In this case, after digging out a tree, the roots are usually trimmed by hand, and after that, the tree is planted back in the field to continue growing [15]. Trees can be planted from the late autumn after the leaves fall to the early spring before the buds break [64]. If a tree is harvested in the fall and expected to be planted in the spring, it has to be stored in a cooler at an optimal temperature and humidity; if not, the tree should not be kept in the cooler for longer than 4 weeks [88]. Trees stored through the winter should be sweated or watered before planting for better post-planting performance. Sweating or watering helps some genera to leaf out when they become intensively dormant while stored in the cooler. The process of sweating takes 3–4 days. It can be performed by placing the tree in the overwintering house or covering it with a few layers of straw or burlap, under which humidity is perfect to bring out the tree from dormancy and force the buds to break; at the same time, the root system should be potted or soaked for several hours. Sweating should be carried out right before planting; trees cannot be sweated in early spring and wait a few months for planting, or be sweated in early spring and planted right away, which can lead to the dieback of new growth [8,89].

**Figure 3:**
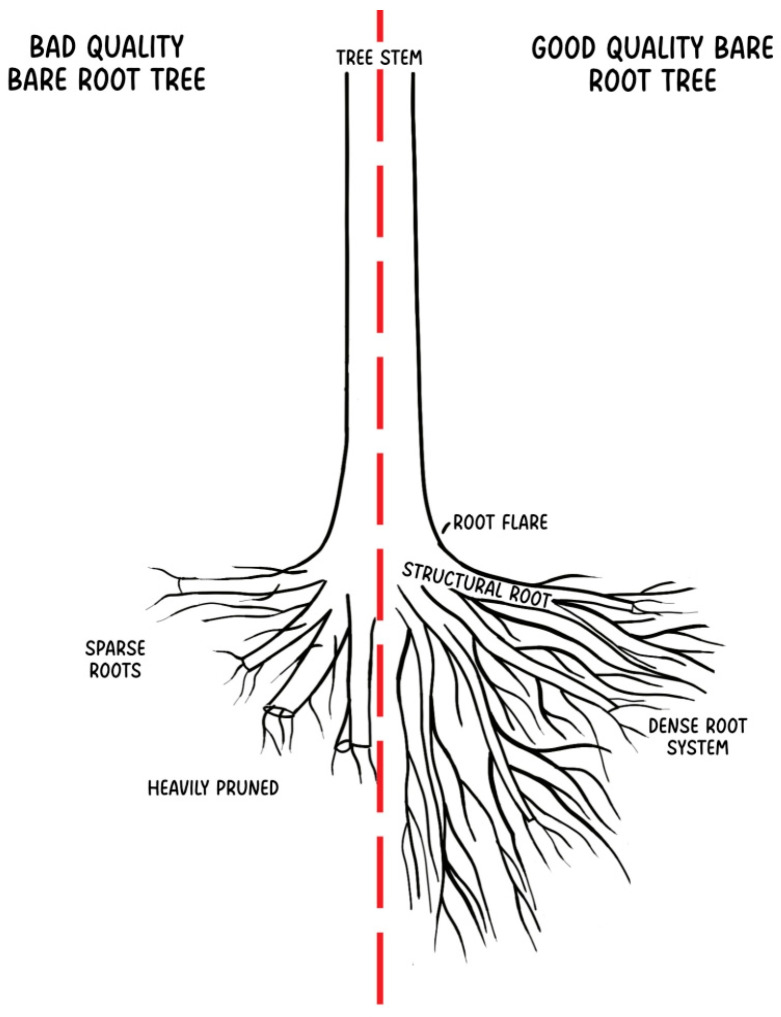
Comparison of bad- and good-quality bare-root trees.

Bare-root trees are characterized by low weight and small size, making them easy to transport and plant, usually needing staking [41]. Due to the exposed root system, roots may dry out during the process if they are not properly secured [17]. Because of that, it must be performed as fast as possible, and any delay may weaken or even kill the tree. Studies indicate that bare-root trees have lower survival rates than container-grown and burlap-wrapped trees [8,90]. If properly watered and maintained, bare-root trees can be a considerably good and cheaper solution for harsh condition sites, where, in a few years, they can reach growth similar to burlap-wrapped trees [91]. Bare-root trees are also available in smaller sizes, but the variability of species and cultivars is limited [17]. Compared to other types of production, bare-root trees are the cheapest material available [43]. A bad-quality bare-root tree is usually heavily pruned with sparse roots. The root system is primarily composed of thick, pruned roots with a minimal number of fine roots. A good-quality bare-root tree is characterized by a pruned but still dense root system with numerous fine roots (Figure 3).

## 3. Tree-Planting Practices and the Following Performance

Successful planting requires knowledge of plant biology and the impact of different habitat conditions on tree growth, including water management [13,36]. Among nursery quality requirements and post-planting tree care, the planting itself is one of the most important actions, which, when wrongly performed, can lead to poor performance of a tree, or even its death a few years after planting, regardless of the quality of the material and its proper tree care, including watering [8,57]. Nevertheless, more research is still needed on production methods and transplant practices to fully recognize recurring problems of newly planted trees and determine possible actions to improve their establishment [17,70,74]. According to Richardson-Calfee and Harris [92], the most favorable planting time for most trees in temperate regions is fall and spring; nevertheless, planting in other terms is possible but leads to additional expenses; thus, planting term is determined mainly by economic factors. Referring to the mentioned planting terms for different types of production, studies show that planting in fall can benefit trees if they are properly and individually watered [90]. According to Good and Corell [93], autumn planting in temperate regions should be carried out at least 4 weeks before the soil temperature drops below 5 °C. Roots can regenerate before low winter temperatures leave trees completely dormant. For trees planted in the spring, there is no significant difference between collective and individual watering [90]. It is essential to know that every species reacts differently to transplanting, and stress effects are usually most exhibited up to three years after planting the tree [81]. Every young tree has to cope with numerous stress factors related to planting, such as water deficiency or poor soil conditions [87,91]. A single stress factor may contribute to the death of a tree; nevertheless, the death is often caused by a complex of stress factors [94,95]. Improper nursery tree production seems to be the main factor connected to tree mortality after planting, and other stress factors are more likely to affect tree growth and vigor—especially profound structural roots [90]. Before planting, a tree should be checked for damage caused during the harvesting and transport and the presence of pests. Removing the tape around the trunk may be necessary to look for holes or stains indicating that the tree is infected. However, it is noticeable that the main issues common for nursery production, as well as further planting and performance, that should be resolved are root defects [16].

### 3.1. Roots Defects

The most critical element that requires assessment before planting is the root system, which should be properly developed and dense, with a rootball not falling apart [49]. Due to the presence of the burlap over the rootball, verification of the condition of the root system may be very difficult or even impossible. Therefore, there is a high risk that the planted tree was not appropriately produced; for example, roots were cut too rarely, leading to the root system being built of only a few thick roots without a dense network of fine roots [15]. It can be partly determined by swinging a standing tree and seeing whether it moves with the whole rootball—when properly rooted or whether the tree moves independently. The rootball remains still when the root system is not dense enough [41]. This problem may also occur when a bare-root tree is attempted to be sold as a burlap-wrapped one, which involves covering the roots with soil and wrapping them with burlap. Unfortunately, many defects cannot be found during the superficial visual inspection due to the possible occurrence of defects in various deeper parts of the rootball, which sometimes may require the removal of a container or burlap and opening it up. One of the most important features of the adequately produced tree is visible root flare; if not, it may indicate future problems with the tree [72].

Many professionals point out that the number of young trees with a root flare located too deep increases; some of them speculate that the problem comes from improper planting, and others say that the main problem is the production process with improper harvesting, making nurseries responsible for defective material [57]. Roots located too deep may experience limited soil oxygen access; their content is lower the greater the depth. Wells et al. [66] indicated that flooding and anoxia at planting sites are crucial for stress increase in newly planted trees—these factors are most significant for roots located too deep. Flooding stress was also studied by Day and Harris [96], which was indicated by lower rates of photosynthesis for *Corylus colurna* L. trees with deep structural roots. During the study, deep planting was not an issue for *C. colurna*, but there was an indication that additional flooding may decrease the vigor of the trees and increase mortality. For many of the species used in urban areas, flooding the root system increased the risk of growth debility for several years after planting or even of death of the tree [97]. This matter may be resolved by using smaller trees with small root systems, which will not be submerged in water at locations where high groundwater levels can be problematic [41]. There are exceptions whereby for some species, especially ones naturally occurring in wetlands, planting too deep may be favorable when transplanted, or at least some species can be tolerant to such conditions, including soil contacting the bark of the stem or burial by additional soil over root system [98].

Unfortunately, in most cases, if the root flare is not visible, the rootball is probably covered with excess soil on top of it, causing deep structural roots [72]. According to Drilias [99], due to improper planting or production processes leading to deep structural roots, trunk diseases like basal canker associated with *Fusarium* and collar rot caused by *Phytophthora* may occur. According to Smiley [100], the most common diseases leading to tree death, which are connected to deep structural roots, are *Armillaria* and *Phytophthora*, particularly effective when infection leads through root collar. Day et al. [98] studied *Quercus alba* L. and *Liquidambar styraciflua* L. bark response to deep planting, where the bark of tested oak trees was rotting and turning black, with three species of saprophytic fungi identified, i.e., *Penicillium*, *Pestalotia*, and *Trichoderma*. In contrast, no fungi were identified on the bark of L. *styraciflua*. The bark decomposition was slow, with no signs of pathogens that may be harmful to the tree. Nevertheless, the process may create favorable conditions for further unwanted infection. Smiley [100] warns about the bark infection process and the need to excavate the trees whenever a deep planting is identified, and is convinced that excess soil abutting the bark can cause premature death of numerous species. If it is 2–3 months since planting, and the tree root flare is located too deep, the best solution is to replant the tree to the correct depth [57]. If the tree is planted longer than that, excessive soil should be changed to mulch. It will degrade slowly, exposing the trunk and accommodating the change [101].

Awareness should be paid to deep structural roots when purchasing trees from nurseries or brokers and during the planting of a tree. Root flare depth can affect tree performance, similar to how the quality of the tree can be an issue, and this applies to both container-grown and burlap-wrapped trees [75]. Naturally growing trees originated from seeds have structural roots near the soil’s surface, usually with no girdling roots or root collar diseases. Trees originating from nurseries cultivated in urban environments may have roots and root collars located too deep, i.e., even 30 cm under the soil surface [99]. Excess soil over the rootball that causes deep structural roots comes from various sources, often from nurseries where additional soil is burlap-wrapped with the rootball or added to the container, but also from planting sites where the tree is placed in too-deep planting pith; or from ongoing construction from which soil may be spread around the tree occasionally [100]. There are many reports about problems related to improper depth of the structural roots (Table 1). Rathjens and Syndor [102] emphasize that the average depth of structural roots in nurseries and brokerage lots in Ohio is about 6 to 8.5 cm. Watson and Hewitt [103] indicate that trees with structural roots located at a depth of 8 cm perform even 50% worse by showing signs of reduced vigor and growth. Bryan et al. [74] showed that live oak *Quercus virginiana* Mill. was affected by planting depth, which appeared with shoot growth reduction, and Wells et al. [66] noticed that one year after planting, deep-planted trees had significantly fewer roots in the upper part of the soil than trees planted at the correct depth. The same study resulted in 50% mortality of *Prunus* × *yedoensis* Matsum. planted 15 cm and 31 cm below grade two years after planting, whereas all control trees survived. Jarecki et al. [104] planted bare-root, three-year-old grafted trees at three different depths: with graft union located 15 cm under the top of the soil, with graft union located at the top of the soil, and with root flare visible. After two years, tree caliper growth was not affected by deep planting, emphasizing that trees were planted in good-quality soil, which probably influenced the results. The authors also noted that the study did not apply to poor-quality urban soils and should not be interpreted as such. Gilman and Harchick [72] indicated that about 75% of roots that emerged from the buried part of the trunk were deflected among tested container trees. Further studies by Gilman et al. [79,105,106] indicated that shaving rootballs of container-grown trees with deflected roots may decrease such defects. Shaving does not impact tree height and trunk diameter [107] but it does significantly increase root area [108]. Bryan et al. [74] noticed that *Fraxinus pennsylvanica* Marsh. planted too deep after two years had a 60% greater death rate; deep planting of a second tested species, *Koelreuteria bipinnata* Franch., resulted in a ca. 10% survival rate. There was little response to deep planting in the first year after planting trees, which was also noticed by Gilman and Grabosky [109]. Studies by Fare [75] showed that not every species may be affected by deep planting. Giblin et al. [110] noticed that among deep-planted trees, two species, i.e., *F. pennsylvanica* and *Malus* ‘Spring Snow’, showed that planting depth had a positive effect on the root volume. Nevertheless, the percent of root volume overall decreased for all tested trees with the increasing planting depth. The study of Bryan et al. [74] indicated that planting at grade or above grade had a 100% survival rate three years after planting and is better than planting below grade; the best planting results were observed for trees planted at grade. Gillman and Anderson [111] studied the same oak species. Liners of the Cathedral Oak^TM^ cultivar were planted at different depths from 0 cm to ca. 12 cm. Results showed that the deeper the tree was planted, the more impaired the caliper growth was; at the same time, height growth was not affected. Giblin et al. [110] reported that mortality increases as the planting depth increases among trees planted at 0 cm, 12.7 cm, and 25.4 cm deep. During their 9-year study of *Acer saccharum* Marsh. and *Tilia cordata* Mill., the highest mortality occurred in the first 5 years after planting. For both species, the number of girdling roots increased proportionally with the planting depth, and linden trees produced a significantly large number of suckers. The same authors indicated that caliper growth was significantly higher for *F. pennsylvanica* and *Q. bicolor* Willd. planted at a grade than 15 cm deeper; two other species (*Betula* ‘Whitespire’ and *M*. ‘Spring Snow’) showed no significant decrease in caliper growth depending on planting depth. Smiley [100] found that trees planted by professionals who had already been growing for about two years had about 13 cm of excess layer of soil and/or mulch over the structural roots. Further studies at the Bartlet Tree Research Laboratory showed that 15 cm of excess soil was deadly to white pine (*Pinus strobus* L.) and caused 30% mortality of willow oak (*Quercus phellos* L.), with about 5% mortality for control trees. Bryan et al. [74] noticed that if container-grown trees were properly irrigated after planting, both exposure to raised rootballs or use of mulching over them did not influence the establishment; the only noticeable problem with raised rootballs after planting was susceptibility to being blown over by the wind.

According to Costello and Day [115], if the material covering the rootball is porous enough, the tree’s growth is not significantly affected. European Tree Planting Standard [64] advises using no more than 5 cm of mulch (10 cm in a dry climate), which should be placed away from the root flare. According to Fare [75], the layer of the mulch is usually 5–7.5 cm thick and covers the root flare. Smiley [100] emphasizes that a significant majority of species used in landscaping are not adapted to increased moisture around root flare or even wet conditions; unfortunately, excess soil over structural roots or even improper mulching may cause constant moisture of the bark, leading to infections, inadequate performance, or even death. Research by Fare [75] showed no development of the adventitious roots on the stem part buried in the soil. The Gillman and Anderson studies indicated the opposite: almost all deeply planted trees developed adventitious roots along the stem part buried in the soil [111]. They also noticed that very young plants may be able to develop such roots, but with age, this ability disappears; it is unknown if this ability may increase the survival rate among young plants. Excavation of deep structural roots is a standard treatment among professional arborists worldwide; unfortunately, even though deep structural roots are a common and widely known problem, trees are still planted incorrectly [100]. To determine the occurrence of this defect, structural roots should be located by using a probe or removing excess soil. The topmost structural roots should be located no more than the depth of ca. 7.5 cm and ca. 7.5–10 cm from the tree stem and should consist of at least two roots. Such placement of the structural roots allows the excavation of the root flare and the planting to proceed [116]. In some cases, removing excess soil may be problematic because, for some nurseries or contractors, taking off the burlap and wire basket is treated as destroying a rootball [117]; in many cases, they defend themselves by stating that the tree will lose warranty or the rootball will fall apart. Nevertheless, taking off the burlap and wire basket will not influence the rootball if the root system is produced correctly and dense enough; in any other case, incorrect tree production may be suspected. Excavating excessive soil and removing roots over root flare during planting may result in 41% more time taken to plant a tree [114].

According to Watson [116], trees with deep structural roots, located deeper than 7.5 cm, should not be accepted for planting because of the reduced height of the rootball, which is inconsistent with the requirements of the nursery stock. After removing excess soil from above the structural roots, a measurement of the rootball height should be taken to decide on rejection. If the tree is accepted anyway, it is worth knowing that the root system will be undersized, and the excavated root flare may be damaged by cold or exposure to the sun; because of that, it is recommended to plant it with a root flare located 2.5–7.5 cm below grade. Broschat [112] demonstrated that the problem of deep planting also applies to palm trees. Planting below the grade was tested on *Phoenix roebelenii*, where it was noted that canopy size growth decreased with increasing planting depth, with a very low survival rate for deeper-planted palm trees. Trees with root flare and structural roots located deeper than the natural location should be adjusted to their original position during replanting or harvest. Otherwise, material leaving the nursery is later unknowingly planted too deep at the planting site, which later leads to poor growth and disease occurrence [49]. This issue also applies to brokers or wholesalers who often sell trees with root flares covered with soil layers [102]. When analyzing the reason why roots are located too deep, the term “deep planting” should not be used because one of the main reasons why many trees grow too deep after planting is nursery production; according to that, it is more reasonable to describe the problem using the term “deep structural roots” [49]. This issue was faced in Warsaw, Poland, in 2023, where 100% of 96 trees that arrived on a planting site had deep structural roots; each rootball was covered with about 10–15 cm of extra soil on top of it (Korbik, unpublished data). Even more worrying is that the deep structural roots problem is noted in many trees growing in urban areas; some reports say that about two-thirds of trees have roots located more than 7.5 cm deep. Unfortunately, it is impossible to be certain about the reason for each case [49]. Rathjens and Sydnor [102] surveyed nurseries and brokers where all trees offered for sale had deep structural roots noted. In the worst case, there was about 9.5 cm of excess soil at nurseries, while the shallowest depth at which structural roots were located was about 3 cm. The same problem was noticed on broker trees, where excess soil reached about 11 cm and 7 cm for trees with structural roots located at the shallowest depth. Accumulation of extra soil on top of the root system not only comes from planting liners too deep but also from cultivating the soil in a nursery, which, over the years, creates a layer of soil that must be removed [16]. The high quality of the soil and good condition of the nursery site let the trees develop and survive with deep structural roots; the problem appears once the tree is transplanted to the harsh urban environment. After that, trees usually struggle, and the survival rate is low [49,75].

### 3.2. Tree Planting and Maintenance

Proper planting and caring for young trees significantly affect their survival and growth. Roots of urban trees are often irregularly distributed, with numerous factors that affect their growth [117,118]. According to Schütt et al. [13], unfavorable soil used for planting may cause growth problems related to water availability. On the other hand, Harris et al. [55] claim that fertilizing trees during planting does not influence post-transplant growth. Mulching significantly influences tree growth and further performance [119], and Arnold and McDonald [120] observed that most natural groundcovers are preferable to bare soil; nevertheless, bare soil is still better than pavement. Watering in the first three years after planting is critical for tree survival [121]. However, the effects of irrigation during the establishment may vary, depending on species and cultivars, which may be more or less drought-tolerant [13,122]. According to Gilman [71], most roots of urban planted trees are located within a depth of 30 cm, and most trees growing in such harsh urban conditions do not produce taproots, which may play a significant role during drought [123]. Taproot production may also be limited by nursery production [124]. Frequent watering is more important for tree establishment than large volumes of water used less frequently; in many cases, recently planted trees should be continuously irrigated daily for up to six weeks [125]. Unfortunately, in many cases, trees are not watered as much as might be expected after planting [126,127].

Planting smaller trees is a good solution because their smaller root systems are more adaptable when planted in a new place. Also, smaller trees grow faster after transplanting and thrive in conditions where larger-sized trees are usually not doing well [54,90,128], which makes smaller trees cheaper to maintain. Small trees establish faster than large trees [77]. The opposite was noticed by Struve et al. [129], who proved that large-caliper trees established faster; nevertheless, they had higher mortality (about 58%) than small-caliper trees (0%). Roots usually take about a year to grow into a balanced size compared to the crown; large trees, of 15 cm caliper and more, usually need three years or more to become established [41]. Studies demonstrate that smaller trees may outgrow large trees in a short period; large trees need about ten years to regenerate 100% of the original root system, while it takes about 5 years for smaller trees [116]. Compared to small-sized trees, large ones need more water, have a larger mulching area beneath them, and sometimes have to be anchored with dedicated tree anchoring systems. All of that initial maintenance increases the final costs of tree planting. Unfortunately, in many cases, produced trees have root defects that affect their quality, initial establishment, and long-term performance on the planting site [49,74]. The connection between improper tree production, planting practices, and further tree establishment is still unknown [129]. Still, tree maintenance techniques are mentioned as part of the survival problem [75,130]. Due to various stresses occurring during the transplanting of a tree and the need to recover the root system afterward, it is not recommended to prune the crown in the first years after planting [15]; first pruning should be performed in the second or third year after planting, and any other pruning before that time should be limited only to removal of broken, dead, or infected branches. The negative effect of crown pruning is indicated by the decrease in radial growth of the trunk [131,132] and the decrease in the vigor of trees, which increases exposure to pathogens and can lead to dieback [133]. Roots of burlap-wrapped trees and container-grown trees are less vulnerable to drying out during transport and before planting because of the presence of the rootball [17]. Nevertheless, there should be as little time as possible from the moment the tree leaves the nursery to its delivery to the planting site, and planting, to encourage less stress during the planting time and better growth of the tree in following years [95].

In contrast to the B&B or container-grown trees, the roots of bare-root trees are exposed during transport and storage, and they are more likely to dry out. Because of that, they should be additionally protected and watered to keep their roots moist [8,17,89]. Plants should be harvested and transported when dormant to reduce the stress associated with water deficiency; if not, ongoing transpiration may significantly weaken the plant, which may be problematic, especially for these species that shed their leaves in late autumn [134,135]. Chemical or hand defoliation may sometimes be performed to force dormancy [136] from the two; the latter is more time-consuming and expensive [134]. Early defoliation affects photosynthesis and starch production, which may result in poor performance of the tree in the following season. For some trees, early defoliation leads to low levels of starch, which will have a negative impact on the growth and vigor of the tree; in other cases, trees that already have high levels of starch may be slightly affected by defoliation [136]. Because of smaller amounts of carbohydrates accumulated in the plant due to early defoliation, the frost resistance may decrease [135]. The later defoliation proceeds, the better for the tree [136]; considering the effectiveness, significant is also the stage of growth of the plant, as well as the number of sprays if the chemical method is used [137]. Bi et al. [138] noticed that some foliage necrosis may occur during chemical defoliation, with green leaves falling as a result; nevertheless, there is no damage to bark, shoots, or buds. Defoliation may take a few weeks after the defoliant application [139].

To prevent additional stresses, the planting pith should be already dug before the arrival of the tree at the planting site so the tree can be planted right away [95]. Generally, untreated burlap decays fast, while treated burlap does not decay noticeably and remains for many years in the soil; synthetic burlap decay is even slower, and, as noted, the burlap may be a serious barrier to roots, and may stop them from growing outside the rootball or can lead to root girdling [140]. The untreated ones do not seem as problematic, so the treated and synthetic burlaps should not be left on the rootball. Nevertheless, it is better to completely remove the burlap when the rootball is placed in the planting pith to avoid performance problems in the distant future [141]. In some cases, the upper part of the burlap is folded back, which supposedly uncovers the rootball and lets the roots grow without any obstruction. Unfortunately, folding the burlap back and not cutting it off leads to the creation of a multilayer of burlap around the rootball, with gaps between, which may prevent the root from growing through it. In this case, cutting the burlap back seems to be the best solution [140]. Completely removing the burlap and wire basket may impact planting time and costs and cause soil cracking and rootball distortion, with no significant difference in twig elongation and caliper size. Trees with the wire basket removed may need staking [142]. Nonetheless, a few additional minutes during planting to localize the root flare and plant the tree at the proper depth may be crucial to tree survival [66], and it saves money that would otherwise be spent on planting another tree once again. Cracking and rootball distortion should not be a problem if the root system is well developed with roots evenly distributed in the rootball; in this case, when burlap with a wire basket or a container is removed, the rootball should stay intact [76]. Removing the burlap and wire baskets should cover as large a part as possible, or at least the top half of the rootball [117]. Burlap and wire baskets should be removed as far as possible, or at least from the top half of the rootball [117]. Other studies indicate that taking off the burlap and wire basket should be a personal choice, based on studies on small-diameter trees at nurseries where they do not meet the same types of stress as in urban conditions [143]. The same problem may occur with wire baskets, which, if left without interference, may cause defects to the roots or even the trunk when the wire is placed too close to it, growing in the following years [142]. Because of that, planting a tree without any interference into a burlap-wrapped rootball seems to be a bad practice, even though it is quite common. Due to the large number of container-grown trees with girdling root problems, it is necessary to check the root flare and structural root condition. If girdling roots are present, they should be cut before planting, which may help prevent future problems [141].

## 4. Conclusions

Greenery has become an important element of cities, and its proper establishment, maintenance, and functioning depend on many factors. Proper identification of these factors and understanding of their impact on trees is the only way to improve the quality of plantings, resulting in a successful extension of urban trees’ lifespan. Correct nursery production practices and practices related to planting and further maintenance seem to be equally important. The basis for young plantings is nursery material, which can be very diverse due to the tree parameters and its production method. B&B and container trees undoubtedly predominate among planting techniques, but there are objections to their quality. Each production method has its advantages and disadvantages, which are related to the root system. Properly produced nursery material should have a dense, properly developed root system that enables proper growth immediately after planting. Unfortunately, in many cases, the root system is characterized by numerous defects; it may not be developed enough, with circling roots or girdling roots, especially for trees growing in containers. Successful planting depends on many variables and can be supported by proper practices that have been more or less developed over the years. It is worth noting that some problems seem to be widely known and still not fully unsolved.

One of the most frequently reported problems is deep structural roots, resulting from covering the upper part of the rootball with excess soil. Deep structural roots are found in many trees, regardless of whether we are dealing with B&B or container trees. Material with such defects intended for planting is usually planted incorrectly; the soil excess over rootball is not taken into account, and as a result, the trees are placed more profoundly in the planting pith than they should be. This improper depth often exceeds 10 cm. Covering the root flare with soil may lead to *Fusarium* or *Phytophthora* infection, leading to the tree’s death. In a more optimistic scenario, trees grow poorly. A poorly developed root system due to the excess of soil above the block must, in addition to normal growth, redirect the roots upwards, towards the surface, where the availability of oxygen and water is the highest; in many cases, such growth leads to the formation of girdling roots around root flare. Unfortunately, most research on deep structural roots concerns small-sized material, not regular-sized trees. Regarding water availability, studies show that proper watering can significantly impact the establishment of trees. However, many factors remain unresolved and leave much room for discussion, including the crown pruning when planting, taking off the burlap from the rootball, or even planting trees of different sizes. Conflicting approaches to planting and maintaining trees often lead to misunderstandings, which clearly indicates that these issues should be further studied. However, of all the unexplored issues, the most discussed for several decades is deep structural roots; its clear impact on trees has not yet been definitively determined. This is important for tree establishment because correcting the mistake of planting them too deep after planting is impossible. This means that trees with too-deep root systems are still in cultivation. Problems resulting from nursery production and planting require further research, and awareness of the problems should be increased among people involved in tree planting.

## Figures and Tables

**Table 1:** Minimum depth of root collar and its influence on the growth of trees of different genotypes.

Species	Minimum Depth of Root Collar (cm)	Effects	Reference
*Acer saccharum*	15	Basal canker and collar root.	[99]
*Phoenix roebelenii*	30	Mn deficiency and lower survival.	[112]
*Quercus virginiana*	There is no minimum; the effect increased to max. 18	Water stress (decreased water potential).	[109]
*Koelreuteria bipinnata*	7.5	Growth reduction and lower survival.	[67]
*Acer rubrum* ‘Autumn Flame’; *Acer rubrum* ‘Brandywine’; *Amelanchier arborea* × *grandiflora* ‘Autumn Brilliance’; *Zelkova serrata* ‘Green Vase’; *Cornus florida* ‘Cherokee Princess’	5	Growth reduction on *C. florida* ‘Cherokee Princess’.	[75]
*Acer saccharum*; *Tilia cordata*	13	Tree mortality in the first year; girdling root increase and greater sucker formation on *T*. *cordata.*	[110]
*Betula platyphylla* × *japonica* ‘Whitespire’; *Fraxinus pennsylvanica*; *Malus* ‘Spring Snow’; *Quercus bicolor*	13	No significant changes; deep-planted *F. pennsylvanica* and *Q. bicolor* had worse caliper growth.	[110]
*Acer saccharum*; *Tilia cordata*; *Celtis occidentalis*; *Gleditsia triacanthos*	There is no minimum depth; the maximum depth was 28	Stem encircling roots and stem girdling roots reported, especially at a depth of 10 cm.	[110]
*Quercus virginiana* ‘SDLN’ Cathedral Oak^®^	There is no minimum; the effect increased to a max 11.5	Decreased trunk development.	[111]
*Fraxinus americana* ‘Autumn Purple’; *Fraxinus pennsylvanica* ‘Patmore’ *Gleditsia triacanthos* f. *inermis* ‘Shade Master’; *Acer platanoides* ‘Emerald Lustre’	15 for graft union; root collar depth unknown	Trees were not significantly affected by planting depth.	[104]
Various species	13	Increased mortality with associated diseases—*Armillaria* and *Phytophthora.*	[100]
*Acer rubrum*; *Prunus × yedoensis*	15	Girdling root increase on *A*. *rubrum*; 50% lower survival of *P*. × *yedoensis.*	[66]
*Lagerstroemia indica* × *faureiei*; *Fraxinus pennsylvanica*; *Nerium oleander*; *Platanus occidentalis*	7.5	Lower survival 3 years after planting.	[69]
*Quercus virginiana* ‘SDLN’ Cathedral Oak^TM^	No minimum; the effect increased to max 19	Circling, girdling, and deflected roots reported reduced growth and survival.	[72]
*Corylus colurna*	15	Girdling root increase; mortality of trees planted on a depth of 30 cm related to flooding.	[96]
*Ulmus parvifolia*	5	Growth reduction.	[113]
*Quercus virginiana* Highrise^®^	Two depths—1.3 and 6.4	Trees were not significantly affected by planting depth.	[108]
Various species	3 (average)	Reduced tree performance of *Acer rubrum*, *Quercus bicolor*, *Fraxinus oxycarpa*, and *Tilia cordata*.	[103]
*Acer rubrum* ‘Florida Flame’; *Ulmus parvifolia*	1.3	Trees were not significantly affected by planting depth.	[114]
*Magnolia grandiflora*	1.3	Trees planted 13 cm deep had more circling roots; other trees were not significantly affected by planting depth.	[106]
